# The effect of director-deputy director promotion focus congruence on team knowledge creation: A social identification perspective

**DOI:** 10.3389/fpsyg.2022.981724

**Published:** 2022-09-20

**Authors:** Xue Yan, Jiakun Liu

**Affiliations:** ^1^School of Economics and Management, University of Science and Technology Beijing, Beijing, China; ^2^Business School, University of International Business and Economics, Beijing, China; ^3^School of Economics and Management, Shandong Youth University of Political Science, Jinan, China

**Keywords:** leading group, promotion focus, director-deputy director fit, team knowledge creation, team identification

## Abstract

As a new leadership style, promotion-focused leadership has attracted the attention of theorists and practitioners. Existing research emphasizes the positive value of director personal promotion focus on team creative behavior while overlooking director-deputy director promotion focus fit. Based on Regulatory Fit Theory and Social Identity Theory, this study explored the effect of director-deputy director promotion focus fit on team knowledge creation and the mediating role of team identification. We used polynomial regression and response surface analysis to analyze the data from 674 questionnaires. The results demonstrate that: (1) director-deputy director congruence in promotion focus is positively related to team identification; (2) under the condition of director-deputy director promotion focus congruence, the level of team identification does not significantly increase when director-deputy director promotion focus rises from “low-low” to “high-high”; (3) team identification plays a mediating role in the relationship between director-deputy director promotion focus congruence and team knowledge creation.

## Introduction and background

With the advent of the knowledge-driven economic age, traditional industries are constantly being transformed and upgraded, and new industries are emerging. Developing new products or services has become the key to business success, and the effective development of new products or services relies heavily on knowledge creation (Nonaka, 1994; Styhre et al., 2002). In this context, the study of knowledge creation, which refers to the ability and process of sharing and integrating knowledge to create new knowledge for use in products, services, and systems, has begun to emerge (Nonaka and Takeuchi, 1995). Teams are increasingly trying to win the favor of companies for their flexibility in form, function, and operation (Chen and Liu, 2022). According to a foreign survey, 91% of top managers believe that “teams are the key to organizational success” (Morgeson et al., 2010). Therefore, the use of work teams for knowledge creation has become necessary (Naseer et al., 2022).

From the overall perspective of team management, the ability of a team to achieve creative results is primarily determined by the director’s traits (Miao et al., 2022; Sørensen et al., 2022). In recent years, regulatory focus has gradually received attention in the field of leadership research as a new perspective in the field of organizational behavior. Regulatory focus is a specific way or tendency people exhibit to achieve their goals. It is divided into two types: promotion focus, which seeks gains, and prevention focus, which focuses on losses (Higgins, 1997). Existing research generally agrees that promotion focus is more likely to lead to creative thinking and innovative ideas than prevention focus (Neubert et al., 2008; Li et al., 2011; Shang and Li, 2015; Tuncdogan et al., 2015). Director promotion focus, as an essential creative goal orientation of the team, will have a significant normative and guiding function in the collective creation of new knowledge in the team (Shang and Li, 2015), which, in turn, will have an impact on the team’ knowledge creation. So, can the director promotion focus better predict team knowledge creation in all cases?

Nowadays, many teams have adopted the model in which the director and deputy director work together to lead the team. The deputy director is the “supporting role” of the director and the “leading role” in the work field he is in charge of. At the same time, he/she plays the leader’s or follower’s role. It is also an essential situational factor that subordinates need to deal with in their daily work. The performance of his role will affect the effectiveness of the leader group and the team. However, the deputy director’s “hands-off” phenomenon often exists in management practice. Academic research on leadership has focused only on a single leadership area, and few studies have combined the role of directors and deputy directors as a whole to explore their impact on the team level. To summarize, this study incorporates deputy director promotion focus into the research model to explore how director and deputy director promotion focus together influences team knowledge creation.

To reveal how the promotion focus of the director and deputy director work together on team knowledge creation, this study will introduce social identity theory to elucidate the mediating mechanism between the two. Social identity theory suggests that differences are the leading cause of differentiation within a team and that asymmetries can undermine intra-team homogeneity, hinder the social identity process, or, worse, will intensify conflict (Tajfel and Turner, 2004). In a team, the leader’s personality traits or role-behavior characteristics are essential information received by the members, and team members will self-define and categorize themselves accordingly (Gao and Zhao, 2014). When director-deputy director congruence in promotion focus, the closer the duo’s pursuit of internal team processes and work goals, the deputy director’s advocacy and modeling of the director’s philosophy will convey to team members a robust conceptualization of common goals, thus making team members more able to perceive team identity and increase their sense of team identification (O’Leary and Mortensen, 2010; Luan and Xie, 2014). In summary, the primary research purpose of this study is to explore the influence of director-deputy director promotion focus fit on team knowledge creation and the mediating role of team identification between them based on social identity theory.

**FIGURE 1:**
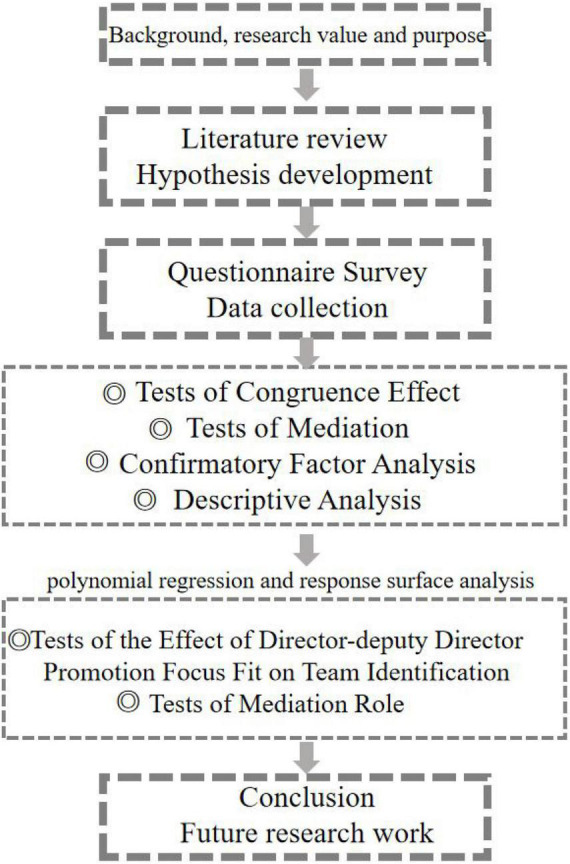
The technology roadmap.

Figure 1 shows the technology roadmap in this article. This article first introduces the background of the topic, then carries on the literature review and puts forward three research hypotheses. Next, this article collects data manually and preprocesses data through a questionnaire survey by using confirmatory factor analysis and descriptive analysis. In the empirical study, this article takes polynomial regression and response surface analysis to study the effect of director-deputy director promotion focus fit on team identification. Finally, the summary and limitations of the study are presented in the last chapter.

## Theory and hypotheses

This study concluded that director-deputy director promotion focus fit could significantly enhance team identification. The concept of team identification is derived from social identity theory, which manifests social identity theory in teams. Team identification refers to the degree of a unified entity within the team and attachment or commitment to team members as perceived by team members (Bezrukova et al., 2009). When an individual identifies with the team, he genuinely cares about the team’s output and image and takes “ownership” of the work to achieve the team’s goals (Van Knippenberg, 2000). Social identity consists of cognition, evaluation, and emotion. Among them, cognition refers to the individual’s awareness of his identity as a group member; evaluation refers to the individual’s assessment of this identity; and emotion refers to the emotional attitude of the group members (Ellemers et al., 1999).

First, director-deputy director promotion focus fit ensures a high level of match between director and deputy director on leadership perceptions, more agreement between them on “change the *status quo* or maintain the *status quo*,” consensus on internal team processes and work goals (Shang and Li, 2015), clearer team boundaries, greater access for members to distinguish their team from other teams, and an enhanced sense of individual identity. Second, according to the social identity theory of leadership, the leader’s group prototypicality is a vivid manifestation of team identification and team values (Van Knippenberg, 2011; Giessner et al., 2013). Director-deputy director promotion focus fit creates a high level of the exchange relationship between the two (Wu et al., 2013), which helps them to be a mutually beneficial relationship, forming a collective leadership synergy and enhancing the leaders’ collective effectiveness. At this point, both the director and deputy director represent the collective interests of the team and can win the trust of team members. The leadership collective is seen as more group prototypes, enhancing members’ evaluation of the team and increasing team members’ collective identification with the team. Finally, by observing the behavioral performance of the director and deputy director, members associate both promotion-focused leadership with team characteristics, see the expectations and requirements of the team, reduce the adverse effects of uncertainty in the external environment, and gain a corresponding sense of purpose. Because of the shared goals, individuals also have a stronger sense of belonging and deeper emotions toward the team and other members (Kim and Vandenberghe, 2018).

Conversely, when the director-deputy director promotion focuses on non-fit, there is a mismatch in how both parties work (Wu et al., 2013). This mismatch reflects the contradiction between the deputy director’s preferred way of working and the director’s personal preference, which can easily lead to cognitive and relational conflicts. In this mismatch, team members may face different “expectations” from the director and deputy director, especially members directly managed by the deputy director, which may weaken the perception of psychological homogeneity within the team. Moreover, these team members may doubt their current behavioral activities and are not sure whether their efforts will be recognized and fairly evaluated by the director. Their distrust of the directors contributes to low team commitment and a common sense of belonging, which is not conducive to forming team identification (Cornelis et al., 2013; Cui and Yu, 2019). At the same time, team members compare and differentiate their differential leadership behaviors by director and deputy director, thus forming subgroups within the team and increasing relational conflict and group political behaviors among members within the team, significantly hindering team identification (Hogg and Terry, 2000). In summary, the level of team identification should increase when the director-deputy director promotion focus fits. Therefore, we hypothesize the following:

Hypothesis 1. Director-deputy director promotion-focused congruence is positively related to team identification.

It includes both “high-high” and “low-low” cases in the director-deputy director promotion focus fit. Research on team process effects suggests that leaders and teams agree on higher levels of essential work characteristics rather than lower levels of agreement; teams are more effective. Some scholars have suggested that this is related to organizational support (Bashshur et al., 2011) and goal attainment (Gibson et al., 2009). In addition, Matta et al. (2015) found that agreement between leaders and subordinates at higher (as opposed to lower) levels of social exchange relationships resulted in higher levels of engagement and organizational citizenship behavior among subordinates. According to these surveys, we also examined the issue of whether congruence in director and deputy director promotion focus at a “high-high” level of promotion focus affects team identification more positively than does congruence at a “low-low” level of promotion focus.

Research has shown that higher levels of promotion focus are positively associated with prosperity, positive behavior, and creativity, among others (Wallace et al., 2016; Zhou et al., 2016). Specifically, for a leading group composed of a director and a deputy director with different levels of promotion focus, the leading group with higher levels of promotion focus leads the team to pursue achievement and development and can influence team identification in two main ways. First, a leading group with higher levels of promotion focus is driven by ideals and ambitions. It conveys positive and powerful hopes and beliefs to employees through their expression of vision. It has been shown that academic motivation and spiritual inspiration from leaders can lead to a stronger sense of meaningful work and organizational motivation and positively affect subordinates’ role identification and team identification (Wu et al., 2015; Li and Mao, 2018). Qiu et al. (2019) verified that spiritual leadership was positively related to the quality of leader-member exchange and team identification. Second, the leading group with higher levels of promotion focus is willing to push for new things to improve the *status quo* and create a team climate for employees to pursue development and change by building team management norms that support innovation (Higgins et al., 2001). According to social identity theory, “exchange” is one of the mechanisms of identity formation. The team gives material and psychological rewards to meet members’ needs, which promotes their identification with the team (Liu, 2009). In teams with higher levels of creative support, leaders provide more resources to match the creative activities of their employees, and their concern, recognition, and respect for their creative activities meet the social needs of employees. For the sake of reciprocity norms, employees will care about the team’s welfare and urge them to integrate their team membership and role status into their social identity.

Whereas leading group with lower levels of promotion focus is not motivated to lead their team in pursuit of high goals and accomplish their tasks primarily by emphasizing task goals, performance evaluation criteria, outputs, and procedures, although related leadership behaviors also play a significant role, activities associated with a leading group with higher levels of promotion focus lead to more carryover effects, such as increased intrinsic interest in subordinates’ work and leader-member exchange, because it more strongly suggests that the exchange between leader and subordinate is motivated not only by traditional instrumental exchanges but also by the construction of higher levels of motivation and ethics (Gottfredson and Aguinis, 2017). Therefore, we hypothesize the following:

Hypothesis 2. Team identification is higher when there is congruence in promotion focus between director and deputy director at higher levels (high-high) rather than at lower levels (low-low).

Finally, this study will further explore the role of team identification in mediating between director-deputy director promotion focus fit and team knowledge creation. Nonaka and Konno (1998) propose the “Ba” theory of knowledge creation, which emphasizes that the dynamics of knowledge creation exist not only in individuals but also in the interactions between individuals and the interactions between individuals and their environment. A specific interpersonal relationship or a common goal can be the mental place of knowledge creation. According to social identity theory, the identity and category of the social group to which an individual belongs is an integral part of the individual’s self-concept. Once an individual identifies with a social group identity, he/she will hope to achieve the need for self-image enhancement through that group identity (Tajfel, 1982). Thus, team identification enables team members to treat team expectations as intrinsic motivation and to be willing to present themselves on behalf of the team, thus demonstrating self-sacrificing and team-oriented behavior (Wang and Howell, 2012).

In teams with higher levels of team identification, team members are motivated to work harder and seek solutions to problems for the sake of the team as a whole, which promotes individual knowledge acquisition and complex and uncertain problem solving (Fisher, 1997), laying the foundation for team knowledge creation. At the same time, team identification is closely related to team members’ cooperative behavior and their behavior of advising others (Richter et al., 2006). Team identification makes team members trust and positively evaluate the members of the team to which they belong. Positive attitudes toward team members can enhance attention to the information provided by others and provide help for others’ information seeking, thus increasing knowledge sharing within the team (especially sharing tacit knowledge) and positively affecting the team’s information integration and innovation aggregation processes. Thus, team identification facilitates the generation of team knowledge creation.

Integrating the above inferences with Hypothesis 1 and 2, this study concludes that director-deputy director promotion focus fit influences team knowledge creation through team identification. When the director-deputy director promotion focus fits, a high match between director and deputy director regarding leadership perceptions and a high level of cognitive congruence between members within the team is guaranteed. The cognitive congruence between the two aspects works together to create high perceptions of team identity and low levels of interpersonal group conflict, shaping a positive team climate; in this case, members are willing to put in more work effort and cooperative behavior, which then promotes team knowledge creation. Conversely, when director-deputy director promotion focuses on non-fit, team members have difficulty forming shared mental models and worrying about interpersonal risks, forming lower team identification and thus having no motivation to invest more information resources in the team, which hinders team knowledge creation. Therefore, we hypothesize the following:

Hypothesis 3. Congruence in promotion focus between director and deputy director has a positive indirect effect on team knowledge creation *via* team identification.

## Methods

### Sample and procedures

The present study conducted a questionnaire survey in 98 operational branches of a large state-owned bank, in which the director (branch head) and all his subordinate employees were surveyed. Data from different sources were collected in a paired manner to avoid the problem of common method bias. Promotion focus was evaluated by the director and deputy director, respectively, while team identification and team knowledge creation were evaluated by all members of the team and aggregated. A total of 98 director questionnaires and 1,080 subordinate questionnaires were administered to all the sites mentioned above. After eliminating the samples that lacked director or deputy director questionnaires and the samples that had fewer than three employee questionnaires, the study finally obtained 57 (effective rate of 58.2%) valid and fitting samples of directors and deputy directors. In the sample, directors had a management range of 7 to 24 subordinates, with a mean of 11.87 subordinates. Of the directors, 27 persons were male, and 30 persons were female; the mean age was 39.39 years (SD = 7.11); 71.9% of directors had a bachelor’s degree or higher; and the mean duration of employment at the outlet was 4.82 years (SD = 4.78, minimum 0.17 years to maximum 21 years). Of the deputy directors, there were 27 male and 30 female; the average age was 34.40 years (SD = 5.82); 89.5% of deputy directors had a bachelor’s degree or above; and the average duration of service in the outlet was 3.78 years (SD = 5.82, minimum 0.20 years to maximum 26 years). A total of 674 valid matched subordinate questionnaires were returned (effective rate of 62.4%), of which 42.0% were male, the average age was 33.08 years (SD = 9.50), and the average length of service at the outlet was 3.75 years (SD = 5.41, minimum 0.16 years to maximum 27 years). There was no significant difference between the gender, age, and length of service of the unselected sample and the valid sample, and there was no non-response bias. The above information indicates that the sample in this study is well represented.

### Measures

The research scale used in this article is based on foreign research results, which were refined and evaluated in detail to determine the most appropriate Chinese items. A six-point Likert-type scale was used, with “1 = strongly disagree, 6 = strongly agree”.

### Promotion focus

The General Regulatory Focus Measure (GRFM) developed by Lockwood et al. (2002) was used to measure promotion focus. Unlike the Regulatory Focus Questionnaire (RFQ) of Higgins et al. (2001), which focuses on past experiences, GRFM is the most commonly used scale, as it expresses the more stable tendencies of individuals. The scale consists of nine questions, such as “At present, my main goal in work and life is to realize my ideals and ambitions.” The average score of the nine questions represents the score of the directors’ promotion focus. The Cronbach’s α score of the promotion focus of the director and deputy director are 0.89 and 0.91, respectively.

### Team identification

The measurement of team identification is based on the organizational identification scale of Smidts et al. (2001). The applicability of the scale has been tested in China (Li and Mao, 2018). The three questions in the scale are selected, such as “I have a strong sense of belonging to this branch (sales department),” and the mean score of the three questions represents the score of team identification. The Cronbach’s α score is 0.90.

### Team knowledge creation

Team knowledge creation is measured with a three-item scale developed by Mitchell et al. (2009). The sample items are “Everyone has come up with some new ideas creatively,” and the average score of the three items represents the score of team knowledge creation. The Cronbach’s α score is 0.94.

### Control variables

Based on previous studies, educational level heterogeneity, years of experience in current positions, gender composition, team size, and team age structure may affect team member communication interactions and team creative behavior (Brooke et al., 2017); therefore, the current study controls for the effects of educational level heterogeneity, mean tenure, gender composition, team size, and team age heterogeneity on team identification, as well as team knowledge creation.

### Data aggregation

In this study, the data on team identification and team knowledge creation were aggregated from the individual data of employees in the network. To test the reasonableness of the aggregation, we calculated ICC (1) and ICC (2), Rug, and ANOVA of the team mean for these two variables. For team identification, Rwg was 0.74, ICC (1) was 0.12, ICC (2) was 0.60, in ANOVA, F (90, 871) was 2.50, *p* < 0.001;for team knowledge creation, Rwg was 0.64, ICC (1) was0.10,ICC (2) was0.54, in ANOVA, F (90, 871) was 2.15, *p* < 0.001. As a rule of thumb, ICC (1) and ICC (1) for team knowledge creation are slightly smaller than the suggested values, presumably due to the small number of employees in some outlets in the sample (Bliese, 1996) but still with strong intra-group consistency. In conclusion, it is statistically reasonable to aggregate the results of team identification and team knowledge creation at the individual level to the team level.

### Analytical strategy

#### Tests of congruence effect

Hypotheses 1 and 2 were tested using polynomial regression and the response surface approach. Polynomial regression was estimated mainly for Equation (1), where M represents the mediating variable team identification, L represents director promotion focus, and V represents deputy director promotion focus. To better explain the results and avoid multicollinearity, the scores of L and V need to be centered. In addition, response surface plots were drawn from the regression coefficients, and significance tests were performed on the graphical indicators to visualize the polynomial regression results.


(1) $$\text{M}={\text{b}}_{0}+{\text{b}}_{1}\,\text{L}+{\text{b}}_{2}\,\text{V}+{\text{b}}_{3}\,{\text{L}}^{2}+{\text{b}}_{4}\,\text{LV}+{\text{b}}_{5}\,{\text{V}}^{2}+\text{e}$$

#### Tests of mediation

To verify the mediating role of team identification, the block variable approach was used in this study. According to Edwards and Cable (2009), the block variable is a weighted linear combination of the quintiles L (director promotion focus), V (deputy director promotion focus), L2 (square of director promotion focus), L × V (the product of director promotion focus and deputy director promotion focus), and V2 (the square of deputy director promotion focus) to obtain a single variable indicating the consistent/inconsistent effect. The significance of indirect effects was then tested by the bootstrap method.

## Data analysis and results

### Confirmatory factor analysis

To ensure that the data have good discriminant validity, this article conducts validated factor analysis on three variables, promotion focus, team identification, and team knowledge creation. In the testing process, four competing models were set (see Table 1), and the results showed that the fit indicators (χ^2^/pdf = 7.93, TLI = 0.93, CFI = 0.945, RMSEA = 0.075) of the three-factor model were statistically significantly better than those of the other competing models (Δχ^2^ = 1223.519, 1190.213, 2177.412, and 3069.410, *p* < 0.001, respectively). The larger indicator of χ^2^/df may be due to the larger sample size, and the other indicators were above the acceptable level, which indicates that the three main constructs in this study have good discriminant validity. Similarly, according to the basic idea of Harmon’s one-factor test, if there is a serious problem of common method bias in the data from the same source, then the one-factor model should fit the data best in the validation factor analysis of these data (Podsakoff et al., 2003). The test results showed that the one-factor model had the worst fit (χ^2^/pdf = 41.77, TLI = 0.608, CFI = 0.664, RMSEA = 0.206), and the three-factor model had the best fit, further indicating that the common method bias problem was better controlled in this study.

**TABLE 1:** Results of confirmatory factor analyses.

Models	Variables	χ^2^	df	χ^2^/df	Δχ^2^	RMS	CFI	TLI	SOME
Three-factor model	PF, TI, TKC	689.906	87	7.93		0.075	0.945	0.933	0.045
Two-factor model	PF + TI, TKC	1,913.425	89	21.50	1,223.519***	0.146	0.833	0.803	0.093
Two-factor model	PF, TI + TKC	1,780.119	89	20.01	1,190.213***	0.141	0.845	0.817	0.096
Two-factor model	PF + TKC, TI	2,767.318	89	31.09	2,177.412***	0.177	0.755	0.711	0.108
One-factor model	PF + TI + TKC	3,759.316	90	41.77	3,069.410***	0.206	0.664	0.608	0.116

*N* = 57 teams. PF = promotion focus; TI = team identification; TKC = team knowledge creation; Δχ^2^ is the result compared with the assumed three-factor model.

***p < 0.001.

### Descriptive analysis

We calculated the means, standard deviations (S.D.), and correlations among study variables using SPSS 25.0. As can be seen in Table 2, gender composition is significantly and positively correlated with team identification (*r* = 0.21, *p* < 0.05) and team knowledge creation (*r* = 0.31, *p* < 0.001). Team identification is significantly and positively correlated with team knowledge creation (*r* = 0.62, *p* < 0.001). These results provided preliminary support for the hypotheses proposed above. We also used the square roots of the average variance extracted (AVE) to examine the discriminant validity further. As shown in Table 2, the square roots of AVE were larger than each construct’s correlation coefficients, ensuring satisfactory discriminant validity. We further used hierarchical regression analysis to test the hypotheses.

**TABLE 2:** Means, standard deviations, and intercorrelations among study variables.

	*Mean*	*S.D.*	1	2	3	4	5	6	7	8	9
1. Educational level heterogeneity	0.32	0.47									
2. Mean tenure	2.18	2.22	0.14								
3. Gender composition	0.45	0.07	0.23^†^	0.11							
4. Team size	11.88	3.77	–0.07	–0.11	0.06						
5. Age heterogeneity	8.85	2.47	0.05	0.19	0.09	0.10					
6. Director promotion focus	4.42	0.81	–0.12	0.09	–0.08	0.06	0.00	(0.89)			
7. Deputy director promotion focus	4.76	0.76	−0.20^†^	0.08	–0.02	–0.05	0.05	–0.06	(0.91)		
8. Team identification	5.09	0.31	0.10	0.04	0.21*	0.14	–0.02	0.12	0.14	(0.90)	
9. Team knowledge creation	4.69	0.39	0.13	–0.19	0.31***	0.09	–0.03	0.00	0.07	0.62***	(0.94)

*N* = 57 teams. Values in parentheses represent coefficient alphas. ^†^*p* < 0.10, *p < 0.05, **p < 0.01. ***p < 0.001.

### Tests of the effect of director-deputy director promotion focus fit on team identification

**FIGURE 2:**
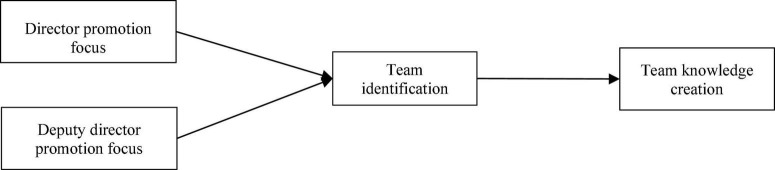
Shows our theoretical model.

Hypothesis 1 predicts that the level of team identity will be higher in the case of director-deputy director promotion focus fit compared to non-fit. To test Hypothesis 1, the present study conducted polynomial regression with team identification as the dependent variable, and the first column of Table 3 shows the estimated coefficients of this regression, as well as the slope and curvature of the consistency line (L = V) and the inconsistency line (L = −V). According to the results of the data in Table 3, the surface along the consistency line (L = V) tends to curve upward (curvature = 0.20, *ns*), indicating that the surface along the consistency line converges to a “U” type. The curves along the inconsistency line bend downward (curvature = −0.25, *p* < 0.05), indicating that the surfaces along the inconsistency line are inverted “U” type. The significant difference in such a direction proves that team identification is significantly higher in the congruent case than in the incongruent case. Figure 2 is a surface diagram based on the regression coefficient. The consistency line in the figure is from the fore-end (L = V = −2.5) to the rear-end (L = V = 2.5), and the inconsistency line is from the left side (L = −2.5, V = 2.5) to the right side (L = −2.5, V = −2.5), the “U” type along the consistency line and the inverted “U” type along the inconsistency line indicate that team identification is higher when director-deputy director promotion focus fit, thus supporting Hypothesis 1.

**TABLE 3:** Polynomial regression results for director-deputy director promotion focus fit.

Variable	Team identification		Team knowledge creation	
Constant (b_0_)	4.82***	4.80***	4.19***	0.62
Educational level heterogeneity	0.06	0.09	0.05	–0.02
Mean tenure	0.01	–0.00	−0.04^†^	−0.04^†^
Gender composition	0.57	0.52	1.09	0.71
Team size	0.02	0.02^†^	0.01	–0.01
Team age heterogeneity	–0.02	–0.02	–0.01	0.00
Director promotion focus (*L*) (*b*_1_)		–0.09	–0.09	–0.03
Deputy director promotion focus (*V*) (*b*_2_)		0.10	0.08	0.01
*L*^2^ (*b*_3_)		–0.05	–0.05	–0.02
*V* × *L* (*b*_4_)		0.22*	0.13	–0.03
*V*^2^ (*b*_5_)		0.02	0.02	0.01
Team identification				0.74***
*R* ^2^	0.09	0.23	0.23	0.49
Δ*R*^2^		0.14*		0.26
Congruence (*L* = *V*) line				
Slope (*b*_1_ + *b*_2_)		0.01	–0.07	–0.02
Curvature (*b*_3_ + *b*_4_ + *b*_5_)		0.20	0.10	–0.04
Incongruence (*L* = -*V*) line				
Slope (*b*_1_ – *b*_2_)		−0.18^†^	–0.12	–0.04
Curvature (*b*_3_ – *b*_4_ + *b*_5_)		−0.25*	–0.16	0.02

*N* = 57 teams. Unstandardized regression coefficients are reported. b_0_-b_5_ corresponds to coefficients in Equation (1).

^†^*p* < 0.10, *p < 0.05, **p < 0.01, ***p < 0.001.

**FIGURE 3:**
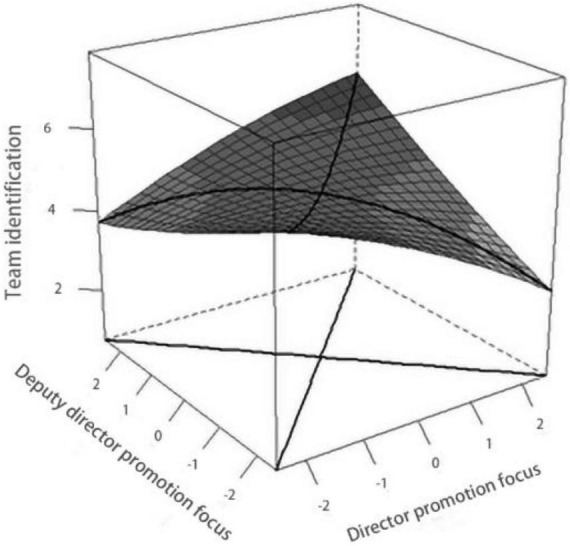
The effect of director-deputy director promotion focus fit on team identification.

At the same time, the slope is not significant (slope = 0.01, *ns*) when it is consistent, indicating that there is no significant difference between director-deputy director promotion focus fit in the cases of “high-high” and “low-low” and the team identification in the case of “high-high” is not higher than that in the case of “low-low,” and Hypothesis 2 was not verified. We can also see this trend in Figure 3, where the front edge points (“low-low”, L = V = −2.5) are essentially parallel to the back edge points (“high-high”, L = V = 2.5) of the surface plot.

### Tests of mediation role of team identification between director-deputy director promotion focus fit and team knowledge creation

In Hypothesis 3, it is hypothesized that director-deputy director promotion focus fit influences team knowledge creation through team identification. In Table 3, team identification positively influences team knowledge creation after controlling for the promotion focus level of the director and deputy director and their polynomials. The block variable approach was used for the analysis to confirm the mediating mechanism. Table 4 reports the results of the mediating effects, with the block variable of director-deputy director promotion focus fit/non-fit being significantly positively related to team identification (path a = 0.39, *p* < 0.01) and team identification being significantly positively related to team knowledge creation (path b = 0.59, *p* < 0.001) after controlling for the corresponding block variable. The product of the path coefficients of the indirect effect, ab, was tested using the bias-corrected non-parametric percentile Bootstrap method, with an estimated value of ab = 0.24 and a 95% confidence interval of [0.0590, 0.4262], which does not contain 0, indicating a significant indirect effect. This result suggests that team identification mediates the effect of director-deputy director promotion focus fit on team knowledge creation, thus validating Hypothesis 3.

**TABLE 4:** Results for indirect effects of director-deputy director promotion focus fit on team knowledge creation.

Variables	Boot a value	Boot b value	Lower interval	Upper interval
Coefficient of the block variable (a path)	0.39**			
Coefficient of team identification, controlling for the block variable (b path)		0.59***		
Coefficient of the block variable, controlling for team identification (c path)		0.09		
Indirect effect (ab) of director-deputy director promotion focus fit *via* team identification.		0.24**		
95% bootstrapped CIs for indirect effect (ab)			0.0590	0.4262

*N* = 57 teams. Standardized coefficients are reported. Bias-corrected confidence intervals (CIs) in 20,000 bootstrap samples are reported.

^†^*p* < 0.10, *p < 0.05, **p < 0.01, ***p < 0.001.

## Discussion

### Results

The existing research emphasizes the positive value of director personal promotion focus on team creative behavior while overlooking director-deputy director promotion focus fit. This article explored the effect of director-deputy director promotion focus fit on team knowledge creation and the mediating role of team identification. Polynomial regression and response surface analysis are used to analyze the data from 674 questionnaires from 57 teams. The results showed that the teams experienced higher levels of team identification when the director-deputy director promotion focus was congruence than when their contrasting promotion focus was. The level of team identification did not significantly increase when the director-deputy director promotion focus rose from “low-low” to “high-high.” Ultimately, team identification mediated the relationship between director-deputy director promotion focus congruence and team knowledge creation. The reason why Hypothesis 2 is not supported may be down to the fact that when director-deputy director promotion focus is at a high level, the leading group has higher expectations for team goals and members, which increases employees’ motivation and satisfies their high-level needs while also expanding the scope of their work and increasing their work tasks, which requires more time and energy on their work and depletes their resources, resulting in a crowding-out effect on team identification, resulting in team identification not being improved accordingly.

### Theoretical and practical implications

This article reveals the influence of director-deputy director promotion focus on team identification and team knowledge creation to make the following contributions. The research extends the scope of analysis examples for researching regulatory-focused leadership and makes supplements for multi-leader practical research. The existing research explored the influence of leadership on subordinates and the whole team, barely from the single leader view, which neglected what influence the leading group with two or more leaders would impose. Although there are a few types of research, they are only in the theoretical stage with rare empirical studies. In addition, the research on leadership behavior according to regulatory focus theory is confined to the view of a single leader. Generally speaking, the influence of leader regulatory focus on employees is resulted from the combined effect of regulatory focus of leadership collectively and fitting multi-leadership regulatory focus. The article takes the leading pattern of director and deputy director as examples to verify the effectiveness of director-deputy director promotion focus fit, “high-high” fit, and “low-low” fit in the team layer and extends leadership research to the multi-leadership layer, which makes up for the deficiency existing in the current studies on leader regulatory focus. The results reveal the significance of the director-deputy director promotion focus fit, which provides a reference for analyzing leadership behavior from a multi-leader view in the coming days.

Furthermore, the research establishes and verifies the interrelationships of director-deputy director promotion focus fit, team identification, and team knowledge creation and observes the intervening role of team identification, which means that director-deputy director promotion focus fit could stimulate team knowledge creation by deepening team identification. It expands the mediating role of team identification and responds to scholars’ call for more attention on the effectiveness of the identification system for leadership effectiveness. Meanwhile, this conclusion also widens the research of team knowledge creation, which refines the research in team identification and team knowledge creation to some degree.

Our study offers implications for practitioners as well. In practice, companies all place a high emphasis on stimulating team knowledge creation to strengthen their competitiveness. The research explores the relationship between director-deputy director promotion focus fit and team knowledge creation based on team identification, which offers companies some ideas on how to promote team knowledge creation. In the first place, the leading body should be aware of group leadership, which means not only should they focus on the influence of their regulatory focus on employees but also pay close attention to matching the regulatory focus of others. Taking the object as an example, the director and deputy director should review the levels of their own and the others’ promotion focus to adjust their leading method according to the others’ conditions to form a good fit and maximize the specific strengths of the leading group. When selecting leading members, the leading group should fully pay attention to fit. It needs to focus on which leadership style could generate better team efficiency and select more effective interactive subjects by observing, interviewing, and testing, all of which could reduce resource consumption in work. What is more, leaders should fully understand the value of team identification and consider all the influence factors of team identification to anticipate better and take control of the psychological course and behavior of team members, promote team knowledge creation, and improve the core competitiveness of the organization. Leaders with a high level of promotion focus should balance the positive and negative effects of high-performance expectations to avoid the crowding-out effect of team identification.

### Limitations and future research

The research indicates the influence of director-deputy director promotion focus fit on team identification and team knowledge creation using multi-stage director-deputy director fit data. Nevertheless, there still exist some drawbacks. Firstly, the research by collecting valid sample data simultaneously represents a cross-sectional study. However, such a data collection method might be unable to inspect the hidden causal relations between variables. To test independent variables, mediating variables, and dependent variables at different times, future research should use the longitudinal study method; secondly, the research explores director-deputy director promotion focus fit only from the view of consistency fit without consideration of prevention focus, and the researches on director-deputy director regulatory fit shall be refined from the view of complementary fit in future; thirdly, only the mediating effect of team identification for director-deputy director promotion focus fit and team knowledge creation is verified in this research, and the future researches may expand related mediating mechanism from other views, such as the social information manufacturing theory. Fourthly, although team identification is subjected to not only the director and deputy director but also other situational factors, such as employees and working conditions, no moderator variable is introduced in this research. In other words, it is necessary to consider the situational factors to research if director-deputy director promotion focus fit could facilitate team identification more effectively. Introducing a moderator would enrich the experimental models and also serve as a supplement for the effect mechanism of the relations between director-deputy director promotion focus fit and team knowledge creation.

## Data availability statement

The raw data supporting the conclusions of this article will be made available by the authors, without undue reservation.

## Author contributions

XY and JL made significant contributions to the study concept and design. XY was primarily responsible for designing the study, collecting and analyzing data, and drafting the manuscript. JL made several revisions and refinements to the content of the manuscript and helped collect some of the data and drafted the manuscript.
